# comparison of postoperative gait parameters after total ankle arthroplasty and ankle fusion: A systematic review

**DOI:** 10.1097/MD.0000000000038727

**Published:** 2024-07-05

**Authors:** Konstantinos Zygogiannis, Georgios C. Thivaios, Anna Kouramba, Androniki Drakou, Konstantinos Vlasis, Panayiotis Panayiotidis, Dimitrios Kalatzis, Dimitrios Koulalis

**Affiliations:** a Laiko General Hospital of Athens – Orthopaedic Department, Athens, Greece; b Blood Unit and National Reference Centre for Congenital Bleeding Disorders, Laiko General Hospital, Athens, Greece; c Department of Anatomy, Medical School, National and Kapodistrian University of Athens, Athens, Greece; d 1st Department of Propaedeutic Internal Medicine, National and Kapodistrian University of Athens Medical School, General Hospital LAIKO, Athens, Greece; e 1st Department of Orthopedics, Medical School, National and Kapodistrian University of Athens, Attiko University Hospital, Athens, Greece.

**Keywords:** ankle fusion, gait analysis, kinematic parameters, kinetic parameters, spatiotemporal parameters, total ankle arthroplasty

## Abstract

Ankle pathology, such as severe arthritis, often necessitates surgical intervention to restore mobility and alleviate pain. Two commonly performed procedures for end-stage ankle disease are ankle fusion (AF) and total ankle arthroplasty (TAA). This review aims to compare the impact of AF and TAA on postoperative gait parameters. An extensive search in PubMed, Scopus, and Web of Science electronic databases was conducted with the use of the keywords (“ankle arthrodesis” OR “ankle fusion”) AND (“ankle replacement” OR “ankle arthroplasty”) AND “gait.” Clinical studies in terms of postoperative gait parameters were included in this review. At least one of the following gait parameters, included in gait analysis, should be researched: spatiotemporal variables and joint kinematics and kinetics. An initial search revealed 221 studies. After the removal of duplicates and screening of titles,10 studies (7 prospective and 3 retrospective case series) were included for qualitative analysis. In the majority of studies, there is no significant difference in spatiotemporal parameters, such as walking speed, cadence, stance duration, step length, and stride length among AF and TAA patients. Postoperative sagittal ankle ROM, mainly maximum ankle dorsiflexion angle is significantly higher in TAA patients, while results concerning hip and knee ROM are variable. The comparison of AF and TAA in terms of postoperative gait parameters has shown variable results. In the majority of studies, there is no significant difference in spatiotemporal and kinetic parameters among AF and TAA patients. Further high-quality prospective studies are needed to fully elucidate the comparison of postoperative gait parameters.

## 1. Introduction

Ankle arthritis is a debilitating condition characterized by the degeneration of the cartilage in the ankle joint, resulting in pain, stiffness, and limited mobility. Two surgical options for end-stage ankle arthritis are ankle fusion (AF) and total ankle arthroplasty (TAA). Both procedures aim to alleviate pain and restore function, but they differ in their approach and outcomes.^[1,2]^

AF involves the surgical fusion of the tibia and talus eliminating tibiotalar movement and creating a solid bony connection. The procedure includes removing the remaining cartilage from the joint surfaces and fixing the tibia and talus bones together using screws, plates, or rods. The bones are compressed to promote bony fusion and bone grafts may be used to enhance fusion rates.^[3]^ AF performed either through an open or an arthroscopic approach, provides excellent pain relief in most cases, with reported success rates ranging from 80% to 90%. The fusion creates a stable and pain-free joint, but it sacrifices ankle’s range of motion (ROM). Patients may experience limitations in certain activities and adaptations in their gait patterns.^[4,5]^

TAA is a surgical procedure that involves the replacement of the damaged ankle joint with a prosthetic implant. Initially, the damaged joint surfaces are removed and a prosthetic implant is inserted, trying to mimic the normal anatomy and function of the tibiotalar joint. The prosthetic components consist of a metal tibial component, a plastic insert, and a metal talar component. The implant preserves joint motion and is designed to provide stability, durability, and long-term function.^[6]^ TAA has shown promising outcomes in terms of pain relief, improved function, and patient satisfaction. The success rates of the procedure vary, with reported survival rates ranging from 80% to 90% at 10 years. However, the longevity of the prosthetic implants is a concern, and revision surgeries may be required in some cases.^[7,8]^

Human gait, the complex pattern of movement during locomotion, is a fundamental aspect of daily life. It involves a highly coordinated sequence of movements, integrating multiple body segments and joints. The main biomechanical components of gait can be classified into the stance phase and the swing phase. The stance phase begins when the heel makes contact with the ground and ends when the toe leaves the ground. During this phase, the body weight is primarily supported by the stance limb, providing stability and propelling the body forward.^[9,10]^ The swing phase occurs when the foot is off the ground, allowing for leg advancement before the next contact. In this phase, the body’s center of mass is repositioned for the next stance phase.^[11]^ Various factors influence gait, including the foot’s contact with the ground, joint movements, muscle activity, and body segment coordination.^[12]^

Gait analysis is a multidimensional and quantitative assessment of an individual’s walking pattern. It involves the measurement and analysis of various parameters related to movement, timing, coordination, and biomechanics during walking. Gait analysis provides valuable insights into mobility, balance, coordination, and functional abilities, aiding in diagnosis, treatment planning, rehabilitation, and research in a wide range of fields.^[13]^ The most common parameters evaluated by gait analysis include spatiotemporal variables (gait speed, step length, cadence, stride length, step width, time and duration of stance and swing phases), joint kinematics (ankle, knee and hip ROM), joint kinetics and ground reaction forces.^[14]^

AF and TAA have different effects on postoperative gait due to their distinct surgical approaches and outcomes. AF eliminates dorsiflexion. As a result, patients compensate by adopting a more pronounced hip and knee flexion during the swing phase of gait. This alteration in gait mechanics may cause a slight limp and a reduced step length.^[15]^ Moreover, the lack of ankle motion affects the propulsion phase of gait, where the foot normally pushes off the ground to propel the body forward.^[16]^ Patients with AF may exhibit reduced push-off power, leading to a less efficient gait pattern. Various gait adaptations, such as wider step width, reduced walking speed, and altered weight distribution, may compensate for the loss of ankle motion. The unaffected limb along with the ipsilateral hip and knee joints are over-stressed, resulting in increase wear and early osteoarthritis.^[17,18]^

Unlike AF, TAA aims to preserve joint motion and restore a more natural gait pattern. It allows for better push-off power during the propulsion phase of gait. Since ankle motion is restored, compensatory mechanisms seen in AF, such as increased hip and knee flexion during the swing phase, are diminished.^[19,20]^ Patients can walk with a more natural stride length and reduced reliance on compensatory mechanisms. With a successful TAA, the load is more evenly distributed across the ankle joint, reducing the excessive stress on adjacent joints like the hip and knee. This can potentially minimize the risk of joint degeneration in those areas.^[21,22]^

The present review aims to gather and compare information from the current literature regarding patients with severe ankle arthropathy, who underwent total ankle arthroplasty or ankle fusion, and analyze what are their effects on spatiotemporal, kinematic and kinetic parameters in order to assist clinicians in decision-making for optimal patient care. Finally another question that can arise from the current studies is how a surgical replacement or fusion of the ankle joint can further affect the gait model and parameters in anatomical areas such as the hip and knee joint.

## 2. Materials and methods

An extensive search in PubMed, Scopus, and Web of Science electronic databases was conducted by the first author in April 2022, with the use of the PRISMA guidelines and the EndNote X3 software (Thompson Reuters).^[23,24]^ The keywords (“ankle arthrodesis” OR “ankle fusion”) AND (“ankle replacement” OR “ankle arthroplasty”) AND “gait” were used.

Clinical studies comparing total ankle arthroplasty and ankle fusion in terms of postoperative gait parameters were included in this review. At least one of the following gait parameters, included in gait analysis, should be researched: spatiotemporal variables (gait speed, step length, cadence, stride length, step width, time and duration of stance and swing phases), joint kinematic variables (ankle, knee and hip ROM), joint kinematics and ground reaction forces. Study protocols without available full text and non-human studies were excluded. Additional excluding criteria included studies in non–English language (n = 4), case reports (n = 2), review articles (n = 21), studies not comparing gait (n = 14), conference Papers, studies with a postoperative follow-up of less than 3 months and a minimum of 5 to 10 patients per variable which we consider to be required to obtain results that are most likely to be both adequate and clinically meaningful.

## 3. Results

**Figure 1.**
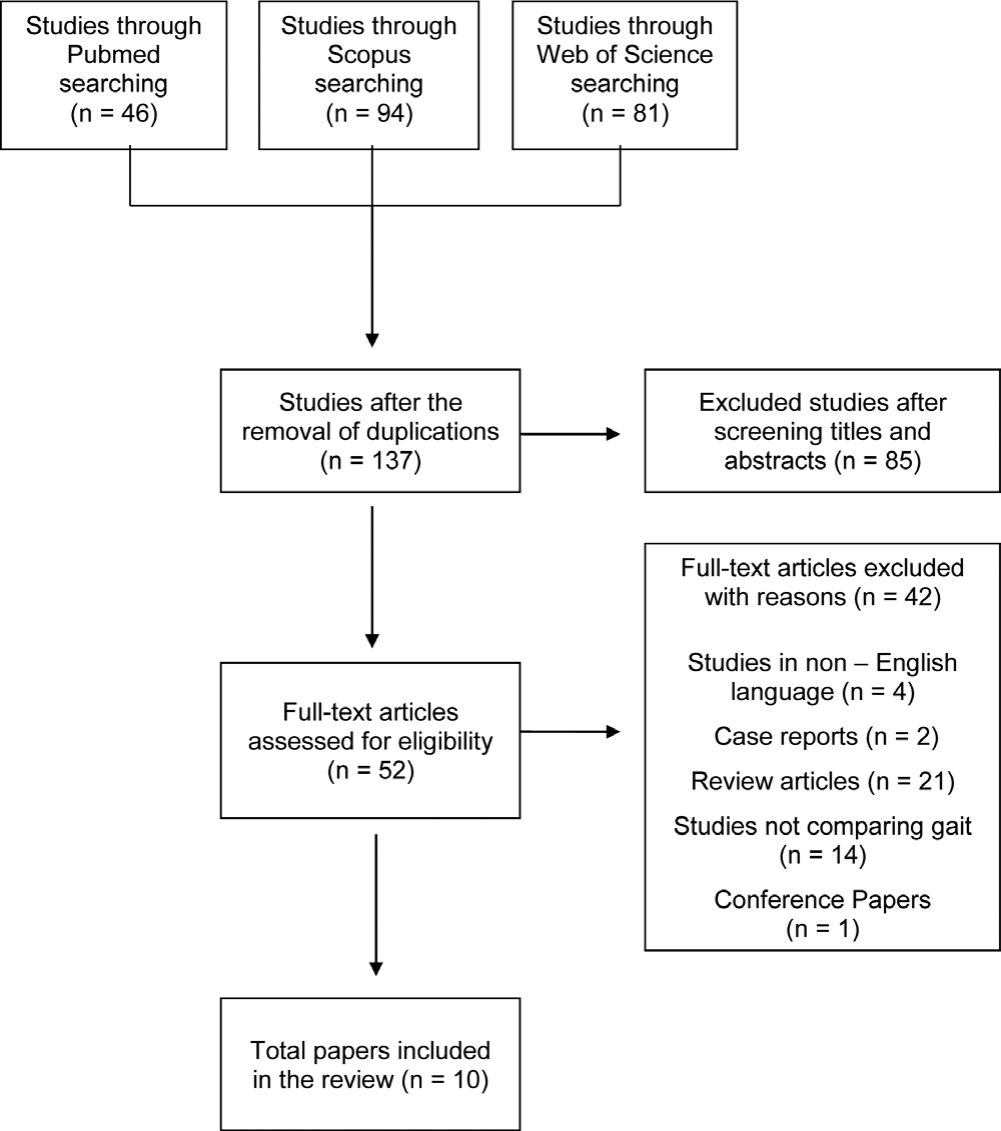
Flowchart of the selected studies.

The initial search revealed 221 studies (Fig. 1). After the removal of duplicates, 137 studies were evaluated. After the screening of titles and abstracts, 85 were rejected, leaving 52 studies for full-text evaluation. Among them, 42 studies were excluded for the mentioned reasons. Finally, 10 studies (7 prospective and 3 retrospective case series) were included for qualitative analysis.^[25–34]^

### 3.1. Spatiotemporal parameters

#### 3.1.1. Walking speed

Walking speed has been evaluated in 8 studies. The majority of them did not find any difference in postoperative walking speed among AF and TAA patients.^[25,27,28,31,34]^ One 3-year prospective and one retrospective study of 12 patients have noticed a higher postoperative walking speed in AF patients, compared to TAA patients. There was no significant difference in cycle length between any of the groups from those 2 studies. However, all intervention groups performed quantitatively worse than the control group, with the arthroplasty groups having shorter cycle lengths compared to the arthrodesis group, though these differences were not statistically significant. In all groups, the duration of the stance phase decreased as walking speed increased. The only significant difference was between the stance phase duration of patients 1 year after arthroplasty (64.1%) and those after arthrodesis (60.2%) at the higher walking speed.^[29,32]^ On the contrary, Seo et al^[33]^ observed a higher postoperative walking speed in TAA patients, compared to AF patients. However, when compared to the control group, gait speeds were decreased in both the TAR and AF groups.

#### 3.1.2. Cadence

No difference in postoperative cadence among AF and TAA patients was observed in 6 studies.^[25,27–29,31,34]^ Two studies have concluded that postoperative cadence was higher in TAA patients compared to AF patients. In the first study by Segal et al, the cadence is increased by an average of 7 steps per minute. These alterations in cadence were consistently observed in each pairwise follow-up comparison.^[32]^ In the second study by Seo et al^[33]^ the mean value of steps per minute was 105.0 ± 10.4 for ankle arthrodesis (AA) while in total ankle arthroplasty, the mean value was 112.0 ± 6.9 demonstrating also an improvement.

#### 3.1.3. Stance duration

In 5 studies, no significant difference was recorded among AF and TAA patients, in terms of stance duration.^[25,26,31,33,34]^ The prospective study by Piriou et al^[29]^ was the only one that observed a higher stance cycle duration in TAA patients in comparison to AF emphasizing that the arthroplasty group exhibits nearly perfect symmetry (with ratios of 1 and 0.97 at 2 different speeds). In contrast, the arthrodesis group demonstrates a shorter duration of stance phase on the operated side, clinically manifested as a limp.

#### 3.1.4. Step length

Postoperative step length was found to be higher after AF, in comparison to TAA, in 2 studies. A significant difference was observed between patients who underwent arthrodesis (S = 1) and those who had total ankle replacement (S = 0.93) in the Piriou et al study, with shorter step lengths noted at both normal and higher speeds. The relative symmetry of step length in each group was expressed as a ratio, denoted as S, with reference to the normal side. A value of 1 indicates that each side is equal in length.^[29,32]^ However, in 2 other studies, no statistically significant difference was noticed.^[25,27]^

#### 3.1.5. Stride length

Four studies have assessed the postoperative stride length following AF or TAA. None of them has found a significant difference in the postoperative stride length among AF and TAA patients.^[25,28,31,33]^

### 3.2. Kinematic parameters

#### 3.2.1. Ankle ROM

Eight studies have compared total ankle ROM in the sagittal plane.^[25,27–32,34]^ In both AF and TAA patients, postoperative ROM has been found to be significantly decreased compared to preoperative ROM,^[25,27]^ as well as in comparison to controls.^[29,34]^ When postoperative sagittal ankle ROM was compared between AF and TAA patients, it was found to significantly increase in TAA patients.^[25,28,31,32]^ Only one study found no significant difference between AF and TAA patients.^[27]^

#### 3.2.2. Maximum ankle dorsiflexion angle

Peak ankle dorsiflexion angle was assessed in 4 studies.^[25,27,31,34]^ One comparative gait analysis study by Flavin et al and Singer et al observed that TAA was associated with higher postoperative maximum ankle dorsiflexion angle, compared to AF,^[27,34]^ while one retrospective study by Sanders et al^[31]^ found no statistical significance. Another retrospective study by Braito et al^[25]^ concluded that maximum ankle dorsiflexion angle was significantly decreased in both AF and TAA patients in comparison to preoperative condition; however, no statistical difference was found among AF and TAA patients, postoperatively.

#### 3.2.3. Maximum ankle plantarflexion angle

Three studies have observed that there is no significant difference in postoperative maximum plantarflexion angle among AF and TAA patients where the range varies depending on the study and the preoperative status.^[25,31,34]^ In AF patients Braito et al^[25]^ reported that the maximum ankle plantarflexion in stance was significantly lower in comparison to the unoperated side. Characteristically, the plantarflexion on the AF side was 6.0(±8.0) degrees while on the non-operative side, it was 4.6(±8.8) degrees.

#### 3.2.4. Total knee ROM

Total knee ROM has been evaluated in 5 studies. Two studies, one prospective and one retrospective with level III evidence, have observed a greater postoperative knee ROM in TAA patients,^[28,33]^ One comparative retrospective study found a greater postoperative knee ROM in AF patients,^[29]^ while two studies, among them one is a 3-year prospective comparative study, found no significant difference among TAA and AF patients.^[25,32]^

#### 3.2.5. Total hip ROM

Three studies were found to assess hip flexion/extension ROM.^[28,32,33]^ In 2 prospective studies, a significant increase in hip ROM was detected after AF in comparison to TAA.^[28,32]^ On the contrary, Seo et al observed that postoperative hip ROM was lower in AF patients, compared to TAA. In this study hip range of motion (ROM) during walking averaged 38.3 ± 9.8 degrees. Both were lower in the AA compared to the TAR, where hip ROM was 45.9 ± 4.6 degrees. In the TAR, the maximum hip flexion was 42.1 degrees (SD 7.4 degrees), and the maximum hip extension was 3.9 degrees (SD 5.0 degrees). In comparison, the AA had a maximum hip flexion of 42.9 degrees (SD 12.1 degrees) and a maximum hip extension of −4.6 degrees (SD 16.5 degrees). The differences in maximum flexion (*P* = .689) and maximum extension (*P* = .194) were not statistically significant.^[33]^

### 3.3. Kinetic parameters

#### 3.3.1. Peak plantarflexion ankle moment

Three studies have observed that there is no significant difference in postoperative maximum plantarflexion angle moment among AF and TAA patients.^[25,31,34]^ Singer et al in a comparative level II evidence study, reported that maximum plantarflexion angle moment in both AF and TAA patients was significantly lower postoperatively, compared with controls. In more detail, the mean plantar flexion motion was 6.2 ± 4.8 degrees for the arthroplasty group and 6.9 ± 6.8 degrees for the arthrodesis group. Both groups had more limited plantar flexion compared to the control group, which had a mean of 16.0 ± 5.6 degrees.^[34]^

#### 3.3.2. Maximum ankle power

Postoperatively, maximum ankle power has been found significantly decreased in both AF and TAA patients, compared to controls preoperatively.^[27,32–34]^ However, maximum ankle power has been found to be higher postoperatively in TAA patients in the 2 studies following studies compared to ankle fusion. Extensively, Seo et al reported that the maximum ankle power in the TAR group (1.16) was significantly higher compared to the AA group (0.32), with a *P* value of .008. Additionally, the comparison of maximum ankle power revealed that the TAR group had a 62.4% deficiency compared to the control group. In contrast, the AA group showed an 89.7% deficiency. Both TAR and AA groups exhibited significantly reduced ankle power compared to the control group, with *P* values of less than .001 and .006, respectively. Moreover, Singer et al suggested that the mean ankle power, along with the standard deviation, was highest in the control group (2.25 ± 0.47 W/kg). In the patient groups, the mean ankle power was lower: 0.69 ± 0.36 W/kg in the arthrodesis group and 0.99 ± 0.69 W/kg in the arthroplasty group.^[33,34]^ Finally in 2 other studies, no statistical significance was detected.^[27,32]^

### 3.4. Vertical ground reaction force

In 2 studies, no significant difference was recorded among AF and TAA patients, in terms of vertical ground reaction force.^[27,32]^ In the same comparative gait analysis study mentioned in other sections by Piriou et al,^[29]^ it is stated that both AF and TAA improved the ground reaction force which appears flattened in the arthritic group. Additionally, it is noted that none of the patients had a ground reaction force identical to the control group. However, the profile of TAA patients was seen to most closely resemble the control group.

## 4. Discussion

Historically, the gold standard of treatment for patients with severe ankle arthritis has been an arthrodesis of the tibiotalar ankle joint, also known as ankle fusion. However, TAA has gained popularity due to significant improvements in implant design and longevity. As TAA has emerged as an increasingly popular surgical option in the treatment of ankle arthritis, understanding the biomechanical differences between AF and TAA is important for improving long-term patient outcomes.^[35]^ The present review has focused on the comparison of gait parameters after AF and TAA.

The history of TAA can be traced back to the mid-20th century when the first attempts at ankle joint replacement were made. However, early designs faced numerous challenges such as implant instability, limited ROM, and high failure rates. As a result, ankle fusion remained the preferred surgical treatment for end-stage ankle arthritis for several decades. The modern era of TAA began in the 1970s with the introduction of the first-generation designs. These early implants consisted of metal components fixed to bone using cement. While they provided pain relief, they were associated with high rates of implant loosening and failure. Despite these limitations, they paved the way for further advancements in ankle replacement technology.^[36]^ In the 1980s and 1990s, second-generation implants were developed, incorporating design modifications to address the issues of implant loosening. These designs utilized more biocompatible materials, such as porous-coated metal and hydroxyapatite coatings, which allowed for better bone integration and long-term stability. However, implant survival rates were still relatively low compared to other joint replacements.^[37]^ The true breakthrough in the evolution of TAA occurred in the early 2000s with the introduction of third-generation implants. These newer designs aimed to replicate the natural anatomy and kinematics of the ankle joint more closely. They incorporated mobile-bearing components, allowing for improved ROM and reduced stress on the implant. Additionally, advances in biomaterials and surface coatings further enhanced implant fixation and longevity.^[38]^ The development of fourth-generation implants included newer designs featured more anatomically accurate shapes, patient-specific instrumentation, and advancements in implant materials. The use of modular components allowed for better customization to fit individual patient anatomy and pathology, leading to improved functional outcomes and patient satisfaction. Recently, there has been a surge in the development of fifth-generation implants, incorporating innovative designs and materials. These advancements have focused on enhancing implant longevity, reducing wear, and improving implant survival rates. Newer bearing surfaces, such as highly cross-linked polyethylene and ceramic coatings, have shown promise in reducing wear and minimizing the risk of osteolysis, a leading cause of implant failure.^[39]^

There are plenty of studies assessing the gait biomechanics following TAA or AF compared to healthy controls. Several studies have concluded that both TAA and AF result in lower gait speed compared with controls while TAA patients exhibit a fairly symmetric gait compared with AF, which requires more compensation through adjacent joints.^[40–44]^ A few studies, highlighted in the present review, have directly compared the effects on gait parameters of TAA against AF.^[25–34]^ However, these studies are characterized by high heterogeneity, low sample size and short follow-up periods.

The study by Piriou et al was the first one to directly compare gait parameters among TAA and AF patients. Authors compared the Salto TAA with AF in 24 patients, with 12 patients in each group. This study observed that neither TAA nor AF restored patients to normal gait but the AF group was associated with faster gait, greater step length, higher knee ROM, and greater asymmetry in gait pattern, while the TAA group exhibited greater stance duration, greater ankle ROM, symmetry in gait, and ground reaction forces approaching those of normal controls. However, the study was underpowered with a short follow-up while the sampling method resulted in different TAA patients being analyzed at 6 months and 12 months postoperatively.^[29]^

Hahn et al compared the gait between 9 AF and 9 Salto TAA patients preoperatively and 1 year postoperatively and found that AF patients had lower ankle ROM with symmetrically decreased dorsiflexion and plantarflexion, whereas the TAA group had an increased sagittal ankle ROM with the largest increase in plantarflexion. Moreover, AF patients exhibited a greater increase in total hip ROM while TAA patients had a greater increase in knee ROM and a trend of increasing ankle power generation during late stance. No differences in spatiotemporal measurements were detected.^[28]^

According to the prospective study by Segal et al,^[32]^ AF patients had a higher step length and walking speed while TAA patients had increased cadence in 1-year and 2-year follow-up. The higher step length of AF patients was accompanied by higher hip ROM, attributed mostly to increased terminal stance hip extension. No difference was found in total knee ROM. It seemed that AF patients took fewer and longer steps with greater hip ROM to cover the same distance compared to TAA patients, possibly at the expense of altered limb loading.

In the study by Seo et al,^[33]^ the gait of 17 TAA patients and 7 AF was compared at 1 year postoperatively. Authors found that TAA patients had a faster gait speed, higher cadence and stride length, increased sagittal ankle dorsiflexion, and greater ankle power, even after height adjustment. No difference was found in stride length, stride time and stance duration. Total knee ROM, total hip ROM and maximum sagittal ankle power was higher in TAA patients.

A prospective study by Flavin et al compared 14 AF patients, 14 TAA patients and 14 normal controls. Authors found no difference in postoperative cadence, step length and walking velocity among AF and TAA patients. Ankle dorsiflexion was higher in TAA patients; however total ankle ROM was similar between the 2 groups. The ankle plantarflexion power and moment were relatively larger in TAA compared to AF. The study was limited by the short follow-up period.^[27]^

A large retrospective study by Braito et al^[25]^ compared 40 AF patients with 101 TAA patients. No difference in spatiotemporal parameters was detected. Mean ankle ROM was 17° after TAA and 12° for AF patients. Similar ankle ROM was reported by the prospective study by Singer et al who concluded that dorsal ankle motion in the sagittal plane was primarily responsible for better gait patterns after TAA. This slight improvement in sagittal ankle ROM after TAA also appeared to be associated with greater knee flexion in stance compared to AF. Authors stated that ankle ROM and forces were not completely normalized by either surgical procedure, but were closer to normal with TAA.^[34]^

The main potential benefit of TAA over AF reported in the literature is the preservation of the existing pre-operative ankle ROM. Even though the ankle joint is fused, some studies have measured ankle ROM.^[27,28,32]^ This reported ankle motion may not be due to true ankle ROM, but may derive from the resulting compensation of the adjacent joints. Although the majority of the studies agree that ankle ROM is increased in TAA patients compared with AF patients, no differences are detected in spatiotemporal parameters, such as gait velocity, cadence, step length and stride length.

It is important to note that individual patient factors, such as preexisting gait abnormalities or muscle weakness, can also influence postoperative gait patterns following AF or TAA. Additionally, postoperative rehabilitation and physical therapy play a crucial role in optimizing gait mechanics and restoring functional mobility in both procedures. Rehabilitation programs focus on strengthening the surrounding musculature, improving ROM, and retraining gait mechanics to achieve the best possible outcomes in postoperative gait.

The present review encompasses some limitations. First, we did not perform a quantitative analysis. Due to the great heterogeneity of the studies, the different gait parameters assessed, the plethora of used implants and the lack of preoperative data, a meta-analysis was not feasible. Most of the studies were prospective cohorts or retrospective case series with a short follow-up and no prospective randomized studies were included.

## 5. Conclusions

The comparison of AF and TAA in terms of postoperative gait parameters has shown variable results. Most studies are characterized by low quality, low sample sizes and heterogeneity. In the majority of studies, there is no significant difference in spatiotemporal parameters, such as walking speed, cadence, stance duration, step length and stride length, among AF and TAA patients. Postoperative sagittal ankle ROM, mainly maximum ankle dorsiflexion angle is significantly higher in TAA patients, while results concerning hip and knee ROM are variable. Most results show no significant difference in postoperative peak ankle plantarflexion moment and vertical ground force among AF and TAA patients. Further high quality prospective studies are needed to fully elucidate the comparison of postoperative gait parameters after AF and TAA.

## Author contributions

**Conceptualization:** Konstantinos Zygogiannis, Dimitrios Koulalis.

**Data curation:** Konstantinos Zygogiannis, Dimitrios Kalatzis.

**Formal analysis:** Panayiotis Panayiotidis.

**Investigation:** Anna Kouramba, Androniki Drakou.

**Methodology:** Georgios C Thivaios, Anna Kouramba, Konstantinos Vlasis, Dimitrios Koulalis.

**Project administration:** Dimitrios Koulalis.

**Supervision:** Dimitrios Koulalis.

**Visualization:** Dimitrios Koulalis.

**Writing – original draft:** Konstantinos Zygogiannis, Dimitrios Koulalis.

**Writing – review & editing:** Konstantinos Zygogiannis, Dimitrios Koulalis.
